# Molecular Assessment of Mating Strategies in a Population of Atlantic Spotted Dolphins

**DOI:** 10.1371/journal.pone.0118227

**Published:** 2015-02-18

**Authors:** Michelle L. Green, Denise L. Herzing, John D. Baldwin

**Affiliations:** 1 Department of Biology, Florida Atlantic University, 3200 College Avenue, Davie, Florida, 33314, United States of America; 2 Wild Dolphin Project, P.O. Box 8436, Jupiter, Florida, 33468, United States of America; 3 Department of Biology, Department of Psychology, Florida Atlantic University, 777 Glades Road, Boca Raton, Florida, 33431, United States of America; The Ohio State University, UNITED STATES

## Abstract

Similar to other small cetacean species, Atlantic spotted dolphins (*Stenella frontalis*) have been the object of concentrated behavioral study. Although mating and courtship behaviors occur often and the social structure of the population is well-studied, the genetic mating system of the species is unknown. To assess the genetic mating system, we genotyped females and their progeny at ten microsatellite loci. Genotype analysis provided estimates of the minimum number of male sires necessary to account for the allelic diversity observed among the progeny. Using the estimates of male sires, we determined whether females mated with the same or different males during independent estrus events. Using Gerud2.0, a minimum of two males was necessary to account for the genetic variation seen among progeny arrays of all tested females. ML-Relate assigned the most likely relationship between offspring pairs; half or full sibling. Relationship analysis supported the conservative male estimates of Gerud2.0 but in some cases, half or full sibling relationships between offspring could not be fully resolved. Integrating the results from Gerud2.0, ML-Relate with previous observational and paternity data, we constructed two-, three-, and four-male pedigree models for each genotyped female. Because increased genetic diversity of offspring may explain multi-male mating, we assessed the internal genetic relatedness of each offspring’s genotype to determine whether parent pairs of offspring were closely related. We found varying levels of internal relatedness ranging from unrelated to closely related (range -0.136–0.321). Because there are several hypothesized explanations for multi-male mating, we assessed our data to determine the most plausible explanation for multi-male mating in our study system. Our study indicated females may benefit from mating with multiple males by passing genes for long-term viability to their young.

## Introduction

Differences in parental investment [1], and ecological factors affecting spatial and temporal distribution of mates and resources play an important role in shaping the mating system of a population [2]. Mating systems develop depending on interactions between costs and benefits of behaviors within and between sexes. From the female perspective, mating bonds in mammalian mating systems include monogamy where females maintain exclusive mating bonds with a single male throughout most of their life, polyandry where females mate with a specific group of the same males in successive breeding attempts, and promiscuity where males and females mate with multiple individuals in successive attempts when no social bond between mating individuals exists [3]. Most mammals have an open polygynous system where one or both sexes mate with multiple individuals [3].

Long-term observations of toothed whales and dolphins (Suborder *Odontoceti*) have led to a better understanding of life history and behavior in these complex and highly social groups [4–7]. In most behavioral studies, male dolphins seek out receptive females, but spend little time with females and new calves [5, 8]. In some locales, males have been observed guarding and coercing receptive females [9] but this observation has not been universal. Because observations typically indicated that males spent little time with females except to mate, mating systems were labeled as promiscuous in species including dusky dolphins (*Lagenorhynchus obscurus* [10]), estuarine dolphins (*Sotalia guianensis* [11]), common dolphins (*Delphinus delphis* [12]), Hector’s dolphins (*Cephalorhynchus hectori* [13]) and bottlenose dolphins (*Tursiops* sp. [9, 14]). Given variable social behaviors and the advancement of molecular techniques, it is now common to distinguish between social and genetic mating systems [15]. The genetic system describes the relatedness of individuals resulting from copulation and breeding, which can differ from the breeding expectations derived from social mating system behaviors such as mate guarding and pair bonding [2, 16]. Discerning between social and genetic mating systems of marine mammals is especially challenging because of the difficulties associated with long-term observation, delayed sexual maturity, long inter-birth intervals and collection of genetic material. Thus only a handful of genetic mating system studies have been completed for cetaceans [17–22].

In this paper we present data on whether observable genetic patterns support a promiscuous mating system, specifically from the perspective of the females. Prior to describing the objectives and methods of this study, it is useful to review the social and demographic factors of the study population that relate to reproductive behavior and provide a basis for a genetic mating system expectation.

### Study population demographics

A well-studied population of Atlantic spotted dolphins (*Stenella frontalis*) has been the focus of intense observational study since 1985 and many demographic and social aspects of the population are well characterized [4, 23–26]. The study site (approximately 480 km^2^) is located on the northwest side of Grand Bahama Island on Little Bahama Bank. The habitat is a shallow sand bank (6–16 m depth) that borders a steep drop off of > 500 m depth into the Gulf Stream. Over the past 25 years, more than 200 individuals have been identified in the study area and the resident population consists of roughly 90–100 individuals each year.

Atlantic spotted dolphins display four developmental color phases [4, 27]. “Two tone” calves (0–3 yr) are born without spots. At roughly three years, dark ventral spots develop and individuals advance to the “speckled” age class (3–9 yr). The “mottled” age class (9–16 yr) begins when white spots form on the dorsal surface and ventral spotting has increased. The oldest age class (“fused”) occurs at approximately 16 years of age and is identified when spots are no longer discrete but “fuse” together into an overall coloration pattern [4].

Many individuals in the study population have been observed for more than 20 years and reproductive histories of females and offspring are well documented. Females become sexually mature near 10 years of age and on average, give birth to a calf every 2.96 years (range 1–5 years [4]). Length of gestation is estimated between 11 and 12 months and two calving peaks (early spring and late fall) have been identified [4]. However, females are not completely synchronized because very young neonates have been observed in August and January.

Both sexes demonstrate natal philopatry and immigration has been consistently low (i.e., only a few individuals per year) for the community [28], indicating a relatively closed population. The sex ratio (M:F) is close to parity with an average ratio across years of 0.97:1 [4, 24]. Age class structure generally consists of 32% fused, 27% mottled and 21% each of speckled and two tone animals [4]. Site fidelity has been relatively high, some individuals in the study population have been sighted regularly for more than two decades [28]. Atlantic spotted dolphins are long-lived and some individuals that were in the fused age class (at least 16 yr) when first sighted in 1985 have been resighted as recently as 2014, indicating an estimated age of at least 45 years.

### Study population social system

Atlantic spotted dolphins live in fission/fusion [29] societies exhibiting dynamic group membership [25, 28]. The study population is considered a single community that is behaviorally self-contained [28] and includes female networks, male alliances, but lacks long-term associations between sexes [25]. The community is subdivided into three social clusters (North, Central and South) [28] consisting of sets of individuals with social associations that are stronger within clusters than between, even though geographic ranges overlap.

Group size in the study population is variable, ranging from 1–60 individuals with a mean group size of nine or fewer animals [28]. Groups may consist of all combinations of sex and age classes, and often consist of associated individuals [28]. Most strong associations occur in same sex groups [25]. Female-female associations are influenced by reproductive status, calf care, and social familiarity [25]. Females associate closely with calves during the first years of the calf’s life [25].

Males form strong associations within and between male pairs/trios that remain relatively constant over time [25]. Males in the study population exhibit two levels of alliance formation; long-term first order alliances and short-term second order alliances which combine two or more first order alliances [25]. Alliance formation is typically attributed to increased female access [30–32] and male Atlantic spotted dolphin alliances have been observed displaying cooperative female tending behaviors [26], although few observations exist. During a cooperative monopolization event in the study community, 3–4 males followed a female, surrounded her, escorted her during feeding and fended off other male groups [26, 33]. Such cooperative events are not necessarily wide-spread among all male alliances nor do these interactions last for extended periods of time. Compared to bottlenose dolphins in Shark Bay, Australia, males may cooperatively tend females for months [9], whereas cooperative tending events in the study population are typically observed over a time scale of minutes to hours and rarely over multiple days.

Although male coalition formation (the joining of forces by two or more parties during a conflict of interests with other parties [34]) is typically attributed to female access benefits [30–32], male alliance and coalition formation are also important in interspecies conflicts in the study population. Sympatric bottlenose dolphins exist in the study area and male Atlantic spotted dolphin alliances form coalitions during agonistic encounters with male bottlenose dolphins [35]. The dynamic interaction between the species is not fully understood but it may be that intraspecies coalitional behavior among Atlantic spotted dolphin males intercepts mating activity between the species and maximizes the reproductive success of potential sires [33].

### What is the genetic expectation?

Most mammalian systems are polygynous and multi-male mating is common [36]. It is expected that females will mate with multiple males potentially due to the benefits of paternity confusion [37], or genetic benefits to offspring [38]. Based on limited genetic investigation, there is evidence that female cetaceans mate with different males throughout their reproductive life. Genetic analysis of humpback whale females and offspring revealed promiscuous mating [17]. In Sarasota, Florida, molecular investigation of bottlenose dolphins (*T*. *truncatus*) indicated that individual males may have sired more than one calf with a given female but a single male had not sired all of the calves from a female [39]. In Shark Bay, Australia, paternity was assigned to different bottlenose dolphin (*Tursiops* sp.) males for two offspring from the same mother [22]. In the study population, mating behaviors have been documented in all age classes and between both sexes [4]. Mating behavior occurs often and it can be difficult to observe all courtship behavior in large groups of animals. Given promiscuous social behavior and evidence from the small amount of literature available, we generally expect females to mate with different males during each estrus event. However, characteristics of the study population raise questions about the validity of that expectation.

In the study population, approximately 100 individuals are sighted each year [40], and the sex ratio is near parity, indicating roughly 50 males in the population each year. Females preferentially copulate with older males [4] and there is genetic evidence that males must reach a minimum age (fused age class) prior to obtaining successful mating opportunities [40]. If only fused males obtain mating opportunities and one third of the males in the community are in the fused age class category [4], then approximately 16–17 males are available for mating. Couple this with the fact that females may avoid mating with some males either because individuals in this small community are genetically related [41] or male quality differs [42], the number of suitable males may be reduced to a small number (< 16). Furthermore, the cohort of suitable males may not change much from one estrus event to the next because the animals are long-lived and immigration in the population is low. Could these factors cause females to mate with the same male during each estrus? Could a low number of males force a promiscuous species into a system where only a few males obtain the majority of mating opportunities? Although the expectation based on socially promiscuous behavior is that females will mate with different males, these concepts raised a reasonable question that has implications for fragmented populations as well as small populations of threatened species.

We tested whether females mate repeatedly with the same male for multiple conception events, or whether females mate with different males. The duration of observation and knowledge of maternal relationships provided a unique opportunity to collect genetic material from sexually mature females and multiple calves, determine whether calves born to a single female were sired by one or more males, and consider the hypotheses that best explain the observed genetic mating patterns. As such our goals were to (1) genetically determine the minimum number of males necessary to explain the allelic diversity of a progeny array, (2) construct pedigrees based on the genetic data, and (3) assess genetic diversity among offspring.

## Materials and Methods

We collected fecal samples from all possible individuals (2000–2007) to address multiple, concurrent genetic investigations of the population. Field seasons consist of approximately 80–100 field days every year from May through September utilizing a 21 m power catamaran as the research platform. Observations and collections occurred from 0700–2000 during days in the study area, with shorter observation times on days of travel to and from the study area or during severe weather. During collection, researchers entered the water for direct observation of dolphins and noted individual information including sex, age class, reproductive status, and behavioral interactions whenever possible. We used underwater observation of spot and coloration patterns as well as nicks, cuts and scars to reliably identify and age individual spotted dolphins. Researchers familiar with the resident population confirmed identifications through digital photographs or video taken of the animals. We compared photos of sampled individuals to a master catalog of all known animals in the population. We assigned unique four-letter identifiers to individual animals and used the identifiers to refer to specific individuals in this study. We based mother-calf assignments on observational data of close association and nursing behavior [4]. We used sighting records to determine the year of birth for each calf and estimated year of conception as one year prior to year of birth. We based the mother’s estimated age at first parturition and age at the birth of each calf on previously reported age estimates [4]. The mother’s age of conception for each calf was based on an estimated 12 month gestation period.

Fecal sample collection, storage and DNA extraction was carried out as previously described by Green *et al*. [43]. We amplified ten polymorphic microsatellite loci to generate genotypes for females and offspring: EV37, EV01 [44], D08 [45], Ttr04, Ttr11, Ttr19, Ttr34, Ttr48 [46], Ttru AAT_44_ [47] and KWM12 [48]. All amplification reactions followed previously reported protocols [EV37, D08 and Ttr48 reported by Green *et al*. [43]; EV01, Ttr04, Ttr11, Ttr19, Ttr34, Ttru AAT_44_ and KWM12 reported by Green *et al*. [40]]. We initially visualized all microsatellite fragments on a 6% polyacrylamide gel stained with ethidium bromide and sized each fragment on an ABI Prism 310 genetic analyzer using Genescan analysis v. 3.1 and Genotyper v. 2.1 (Applied Biosystems, Foster City, California, USA).

Because the current study was part of a larger study, the population-level data set yielded sample sizes appropriate for checks of heterozygote deficiency, deviations from Hardy-Weinberg and genotyping error as a result of null alleles, large allele drop-out and stutter bands. We tested for deviation from Hardy-Weinberg equilibrium and linkage disequilibrium using Fisher’s exact tests and the Markov chain method (10,000 dememorization steps, 1,000 batches, and 10,000 iterations per batch) using Genepop v. 3.4 [49] and Bonferroni corrected for multiple comparisons. We constructed input files for Genepop using Convert v. 1.31 [50]. We calculated allele frequencies, number of alleles per locus, estimates of null allele frequency and polymorphic information content using Cervus v. 3.0.3 [51–53]. We used Micro-checker v. 2.2.3 [54] to assess genotyping error as a result of null alleles, large allele drop-out and stutter bands. In addition, because noninvasive tissues may lead to genotyping errors [55, 56], we took specific precautions throughout the project to reduce genotyping error. We completed multiple amplifications based on the multiple tubes approach [56, 57] for those samples containing low quantity or excessively degraded DNA. We generated a consensus genotype from all amplifications and used the consensus in subsequent analyses. We used Genotyper v. 2.1 to assign allele sizes and all chromatograms were checked by eye to verify the allele sizes. We estimated the genotyping error rate in the data set by a double-blind study. An individual without prior knowledge of the sample genotypes randomly selected seven samples (approximately 54% of the female-offspring subset) for blind analysis. The blind study samples followed the same protocol used to generate the original genotypes in the data set, in that questionable amplifications were re-amplified until a consensus was reached. Questionable samples were re-tested if allele amplitude was close to the baseline or if differences in peak sizes were unclear.

To evaluate the power of our microsatellite loci to detect multiple paternity, we ran simulations in Gerudsim2.0 [58]. Gerudsim simulates sets of offspring genotypes based on specified progeny sizes, draws a sample of offspring from population level observed allele frequencies and estimates the number of sires present in each array. The program used a single multilocus maternal genotype and 1–4 randomly generated paternal genotypes (based on the potential number of male sires in a progeny array of 2–4 calves) to evaluate the probability of correctly determining the number of fathers within an array. Our simulations consisted of individual runs of 2–4 offspring per array generated from 1–4 fathers (100 iterations per run). Exclusion probabilities were generated by Gerud2.0 [58] for array comparisons based on all ten loci when the mother’s genotype was known.

Within the female-offspring data set used for this study, we tested for evidence of multiple paternity using Gerud2.0 which uses a computer algorithm to reconstruct parental genotypes based on multi-locus data from a progeny array when the genotype of one parent is known. Because microsatellite inheritance is biparental, the maternal allele was first subtracted, leaving the paternally inherited allele at each locus for each calf. In some cases, the mother and calf carried identical alleles, meaning the paternal allele could be either allele size. The possible paternal alleles at each locus were combined to generate all possible paternal genotypes for each calf. The reconstructed genotypes were then compared to the progeny array to determine the minimum number of males necessary to explain the alleles in the offspring array. Multiple paternity was assigned as a result of more than two different paternal alleles found among the offspring at a given locus.

To determine whether half or full sibling relationships were more likely between offspring pairs, we analyzed calf arrays using ML-Relate [59]. We estimated population allele frequencies using Cervus v. 3.0.3 [51–53] and all available animals genotyped for the larger, concurrent study. We limited relationship matrix analysis to only the progeny arrays in the dataset. The analysis reported the most likely relationship category (unrelated, parent-offspring, half siblings or full siblings) and specific hypothesis tests were also run to determine whether a half or full sibling relationship was significantly more likely (based on 10,000 simulations).

Based on male sire estimates and previous paternity assignments reported by Green *et al*. [40], we constructed hypothetical pedigrees. Pedigrees for each female and offspring array were constructed for the minimum and maximum number of males. If paternity was previously assigned for any offspring in an array, the father was included. We estimated male age at time of conception using previous analysis [40]. If the exact year of birth was unknown, a minimum age was calculated based on age class and the number of years sighted. For example, if an individual first sighted in 1985 was in the fused age class, their age range in 2009 was estimated as 40+ years because the individual was at least 16 years old in 1985, plus an additional 24 years to 2009. We used field sighting data to consider whether successful male sires (as indicated by paternity analysis) were available for mating during conception years of sibling offspring in each array.

We calculated three microsatellite measures of genetic diversity to reflect the levels of individual inbreeding as a result of closely related parents. We tested standardized multilocus heterozygosity (SH), internal relatedness (IR) and homozygosity by loci (HL; [41, 60, 61]) for all calves using the IR macroN3 developed by W. Amos (http://www.zoo.cam.ac.uk/directory/william-amos). Aparicio et al. [61] suggested that HL performed the best of all three methods, however, we present all three for comparison to other studies. Internal relatedness is similar to Queller and Goodnight’s [62] measure of genetic relatedness (*R*) between two individuals but looks within a single individual’s genotype to compare two alleles at a single locus rather than compare two pairs of alleles. The value is calculated over all loci; negative values indicate outbred parents, zero values indicate unrelated parents and positive values indicate related parents. SH reflects heterozygosity scores at each locus that are weighted by the overall heterozygosity at the locus and HL goes even further by weighing the contribution of each locus to the homozygosity index depending on allelic variability, giving more weight to more informative loci. HL values vary from zero (all loci are heterozygous) to one (all loci are homozygous) and intermediate values depend on the expected heterozygosity levels.

### Ethics statement

The field work conducted for this study was approved and completed under a research permit granted by the Bahamian Department of Fisheries. The Institutional Animal Care and Use Committee at Florida Atlantic University reviewed all protocols and approved the field work for this study.

## Results

Fecal samples were collected from 85 (35 male, 50 female) individual Atlantic spotted dolphins. Tests of multiple paternities among calves from a single mother required genetic samples from females and at least three of her offspring. Three females with at least three offspring each were successfully sampled for this study. Females Flyi and Lgsh were sampled, each with three offspring, and female Pain was sampled along with four calves. Estimated age of first parturition ranged from 10–12 years and birth ages ranged from 10–27 years (Table 1).

**Table 1:** Measures of multiple paternity and parent relatedness for progeny arrays of three Atlantic spotted dolphin females (*Stenella frontalis*).

			Gerud2.0	**ML-Relate**			
Name	YoB	M_B_	Min. no. males	Min. no. males	IR	SH	HL
Flyi							
KP	1992	12*	2	3	0.403	0.564	0.771
Flam	1999	19			0.528	0.376	0.755
Free	2002	22			-0.111	1.128	0.384
ave					0.273	0.689	0.637
Lgsh							
Lhal	1993	12*	2	2	-0.276	1.316	0.269
Lagu	1997	16			0.030	0.940	0.427
Lhas	2001	20			-0.160	1.128	0.309
ave					-0.136	1.128	0.335
Pain							
Brus	1990	10*	2	2	-0.022	0.940	0.506
Pigm	1994	14			0.538	0.376	0.789
Pica	1999	19			-0.020	0.940	0.474
Port	2007	27			0.787	0.188	0.883
ave					0.321	0.611	0.663

Year of birth (YoB), estimate of mother’s age at time of birth of young (M_B_).

* mother’s estimated age of first parturition (mother’s ID in bold, followed by the ID of her calves)

Minimum number of males required to account for progeny array, internal relatedness (IR), standardized heterozygosity (SH) and homozygosity by loci (HL), average of offspring array (*ave*).

All samples were successfully extracted and subsequently amplified across all ten loci. Among all samples included in the blind study, the replicates resulted in allele typing that matched with 100% accuracy to the previously assigned genotypes, providing an estimated genotyping error rate of 0%. There was no evidence of heterozygote deficiency, deviations from Hardy-Weinberg equilibrium, no evidence of null alleles, allelic dropout or genotyping error as a result of stutter bands. The number of alleles per locus for the ten loci ranged from 2–6 (mean = 4.3) and the mean polymorphic information content was 0.49. The overall exclusion probability based on all ten loci combined was 0.98 when the mother was known.

Gerudsim reconstruction accuracy varied with the number of fathers and number of offspring allotted to each generated paternal genotype. In 100% of simulations testing 2–4 offspring from a single father, the reconstructed number of fathers matched the real number. Accuracy was reduced to 65% when testing four calves from two fathers and further reduced to 42% when testing three offspring from two fathers. In simulations testing three offspring from three fathers and four offspring from three or four fathers, the program always underestimated the number of fathers necessary to explain the progeny array. In no circumstances was the number of real fathers overestimated by the heuristic search algorithm utilized in Gerudsim.

Using ten polymorphic loci in Gerud2.0 analysis, it was determined a minimum of two males was necessary to explain the progeny arrays of all three females (Table 1). Analysis with ML-Relate categorized all offspring of Flyi as half siblings, indicating a different male sired each calf. ML-Relate identified one full sibling pair (Lhal – Lhas, full sibling *p* = 0.0344) and two half sibling pairs (Lhal – Lagu, Lhas – Lagu) among the three offspring of Lgsh, indicating two potential fathers. Among the four offspring of Pain, ML-Relate was unable to resolve a series of plausible relationships and, as a result, was unable to determine if two or three males best explained the offspring array. One calf, Pica, was more likely to be a half sibling to all other offspring (mean *p* = 0.032). Brus – Pigm (*p* = 0.041) and Pigm – Port were more likely to be full siblings (*p* = 0.041). However, the relationship between Port and Brus could not be resolved (half sibling *p* = 0.145, full sibling *p* = 0.400).

**Fig 1:**
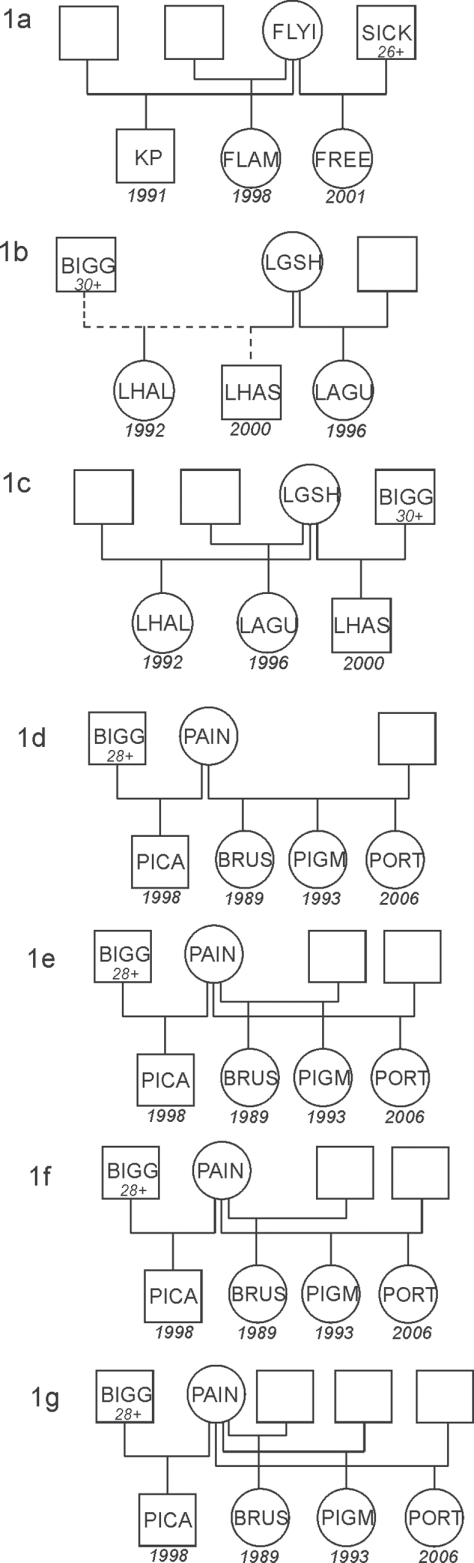
Hypothetical pedigree construction of Atlantic spotted dolphin (*Stenella frontalis*) families based on the integration of previous paternity analysis, observational data and results of Gerud2.0, which estimates the number of males necessary to explain the allelic diversity of the progeny array, and ML-Relate, which determines the most likely relationship (i.e., half or full sibling) between offspring pairs; (a) three-male model to explain the progeny array of female Flyi; (b & c) two- and three-male models to explain the progeny array of Lgsh; (d – g) two-, three- and four-male models to explain the progeny array of female Pain.

Because ML-Relate indicated half-sibling relationships among all three offspring, we constructed a three-male model pedigree for Flyi (Fig. 1A). Based on previous paternity assignments [40], Flyi mated with Sick in 2001 to produce Free. Sick was estimated to be 26 or more years old in 2001. Siblings KP and Flam were conceived in 1991 and 1998, respectively. Sick was first observed in the study population in 1991 and re-sighted every year through 2002, except 1998.

We constructed two-male and three-male model pedigrees for Lgsh. Lgsh mated with Bigg in 2000 to produce Lhas. Bigg was estimated to be at least 30 years old in 2000. ML-Relate indicated a full sibling relationship between Lhal and Lhas (Fig. 1B), however, previous paternity assignments did not support Bigg as the most likely father of Lhal. The three-male model reflects a half sibling relationship between Lhal and Lhas, rather than a full sibling relationship (Fig. 1C). Estimated conception years of siblings Lhas and Lagu were 1992 and 1996, respectively. Bigg was first sighted in the study population in 1986 and re-sighted every year until 2012, except 2007.

We constructed two-, three- and four-male model pedigrees for Pain. Pain mated with Bigg in 1998 to produce Pica, siblings Brus, Pigm and Port were conceived in 1989, 1993 and 2006, respectively. Bigg was estimated to be at least 28 years old at the time Pica was conceived. As mentioned previously, Bigg was first sighted in the study population in 1986 and re-sighted every year until 2012, except 2007. ML-Relate could not resolve the relationship between Pigm and two siblings, Brus and Port. A two-male model reflects full sibling relationships between Brus, Pigm and Port (Fig. 1D). A three-male pedigree represents both scenarios where either Brus and Pigm (Fig. 1E) or Brus and Port are true full siblings (Fig. 1F). If neither of the full sibling relationships is true, a four-male pedigree best explains the array (Fig. 1G).

The average heterozygosity measure within each offspring array varied (Table 1). The genotype of Flyi was similar to those of the males that sired two of her offspring (KP and Flam, HL = 0.771 and 0.755, respectively), whereas the sire of offspring Free was less genetically similar (HL = 0.384). The offspring of Lgsh were more heterozygous (mean HL = 0.335) than the offspring of Flyi (mean HL = 0.637), indicating that the male sire genotypes were less similar to Lgsh than the males that successfully mated with Flyi. Among the four offspring of Pain, two calves had relatively low heterozygosity (Pigm HL = 0.789, Port HL = 0.883) while the other two calves indicated more heterozygosity (Brus HL = 0.506, Pica HL = 0.474).

## Discussion

We confirmed that multiple paternity was common in Atlantic spotted dolphins. Given observational data and the fact that our result was consistent across all three females, this pattern likely represents the majority of the population. Interestingly, ML-Relate indicated the possibility of full sibling relationships rather than half sibling relationships as might be expected in a promiscuous system. It is important to resolve full versus half sibling relationships because each scenario has important and unique implications for the mating system. Inclusive fitness theory proposes that individuals will aid genetic relatives as a mechanism to increase their own fitness [63]. Inclusive fitness benefits may influence alliance formation because male alliances are thought to function in mate access. Relatedness as a factor in male alliance formation is variable among populations. Male alliances in bottlenose dolphins studied in Sarasota, Florida, do not appear to be based on relatedness [64] whereas male alliance members in both Shark Bay, Australia [65] and the Bahamas [66] were closely related. It is not known whether Atlantic spotted dolphins form male alliances based on relatedness. Although full siblings Atlantic spotted dolphins are unlikely to be alliance members because of age differences, preferential coalition formation between alliances containing full siblings may provide inclusive fitness benefits if coalitions function in increasing mating access to females.

Conservative male sire estimates provided by Gerud2.0 were supported by full sibling relationships in ML-Relate. However, Gerudsim2.0 often underestimated the true number of males raising concern that ML-Relate also underestimated the number of half sibling relationships among offspring. Careful consideration is necessary prior to accepting a full sibling pair over a half sibling pair. Available methods for estimating relatedness have a tendency to mislabel relationships [67]. Csilléry et al. [68] found that dyads of previously known pedigree relationships were often misclassified when based exclusively on genetic information. In fact, Csilléry et al. [68] found consistently high misclassification rates; relationships were overestimated and dyads were classified as closer kin than they actually were. In this study population, Green et al. [40] reported overestimates of related dyads among known maternal half siblings based on relatedness analysis that assigned an average of nearly six additional maternal half siblings per individual. It is possible that the full sibling relationships in the current offspring arrays were also overestimated and full sibling relationships may not be true.

Overestimates of close relationships likely stems from low allelic diversity. The population of Atlantic spotted dolphins consists of approximately 90–100 individuals per year. The animals are considered resident and stable with discovery rates indicative of a relatively closed population [69]. Given these factors, it is not surprising that allelic and genetic diversity among individuals was relatively low. A study of Atlantic spotted dolphins encompassing a much larger geographic scale averaged 11 alleles per locus (range 7–15; [70]) compared to 4.3 in the current study population. Given low allelic diversity, similar genotypes may arise in non-related individuals. Consider a scenario where two males with similar genotypes each sire a calf with a single female. Because the males share alleles common in the population, those alleles may assort into similar patterns in two offspring, resulting in *R* values that incorrectly indicate full siblings rather than half siblings. Although it is not impossible for full siblings to arise in small populations, these close relationships are likely overestimated in the current study.

Whether we accept full or half sibling relationships, more than one male was necessary to explain to the progeny arrays. Given the fact that the number of suitable males may be low, the reason(s) why females mate with different males are likely important. One reasonable consideration to explain why females mate with different males is that the same male was not available for subsequent mating. Female Atlantic spotted dolphins reach sexual maturity between 8–11 years of age [4]. Gestation lasts approximately one year and females care for calves for 2–3 years, resulting in a typical calving interval of three years [4]. Therefore, the same male must be available for several years to mate with the same female when she is receptive. While this study did not assign paternity to specific males, paternity was previously assigned for one calf in each progeny array [40]. In nearly all cases, the same male was sighted during the year each female was in estrus. An exception occurred in 1998 between Sick and Flyi. Sick was not sighted in 1998 when Flyi conceived Flam. However, it is important to note that a gap in sighting does not definitively indicate that the individual was not active in the study population throughout a given year. It is possible that Sick was active but simply not observed, making him a potential mate for Flyi. However, it is also possible that Sick was not available as a mate option to Flyi. Overall, sightings suggest the same males were available for mating throughout several estrus cycles but the females did not repeatedly mate them.

If the same male was available for repeated mating after a female successfully raised a calf, why wouldn’t the female mate with the male again? It is important to note that females will not benefit unreservedly from promiscuous behavior. For example, if paternity is confused, females will not receive help from males in raising offspring [4, 33]. Second, if females actively seek out multiple males with which to mate as has been reported for bottlenose dolphins in the Bahamas [71], they may incur energetic costs of travel and costs associated with increased predation risk [72]. Females also increase their chance of contracting a venereal disease [72] such as genital lesions, papillomas [73], or squamous cell carcinomas [74]. What benefits outweigh costs incurred from mating with multiple males [38, 72]? We offer discussion on the most plausible hypotheses for the species.

Hrdy [37] first postulated that females mate with multiple males to confuse paternity and avoid infanticide. The hypothesis fits well in species that give birth to altricial young and where killing offspring results in a mating opportunity for the male [36]. If females are using multi-male mating as a tactic to confuse paternity and therefore avoid infanticide, we expect males to exhibit guarding behaviors to ensure access to the female when she is sexually receptive following the death of her calf [3, 75]. Although cetaceans give birth to relatively precocial calves [76], violent interactions resulting in cetacean infanticide, although rare, do exist. Patterson *et al*. [77] suggested infanticide among bottlenose dolphins in Moray Firth based on a large proportion of stranded juveniles exhibiting injuries consistent with bottlenose attack. Similarly, Dunn *et al*. [78] reported stranded calves died from blunt-force trauma consistent with bottlenose attack. The only reported observation of a violent encounter that appeared to lead to the death of a calf occurred among tucuxi dolphins (*Sotalia guianensis*) [79]. In our study population, no violent actions towards calves have been observed over 26 field seasons. Overall, several independent research groups observe a variety of dolphin behavior in unique populations, and if infanticide was common, we expect more reports.

Furthermore, it is unclear whether male Atlantic spotted dolphins will gain a mating opportunity following the loss of a calf. The average interbirth interval of female Atlantic spotted dolphins with successful calves is 3.56 years, but females that lose a calf significantly decrease their interbirth interval and become pregnant the same or following year [4, 80]. Previous research identified a spring and fall calving peak in the study population [4], indicating that synchronized estrus in the wild may limit female receptivity following the loss of a calf. However, isolated observations of young neonates during off-peak times have occurred and may indicate female receptivity, although it is unknown whether these calves were born to females that previously lost offspring. In Shark Bay, Australia, female bottlenose dolphins that have lost calves became receptive within a few days (based on attractiveness to males; [9]) but it is not known whether the same is true for Atlantic spotted dolphins. When females become receptive, male behaviors such as herding and tending are expected in order to control the mating opportunity. Among Atlantic spotted dolphins, sporadic events of female herding have been reported [26, 33].

In addition to the published reports that may indicate males attempt to control mating opportunities, males have also been observed spending time with very late gestation females and females with new calves. While it does not completely rule out the possibility that males are attempting to control mating opportunities, the lack of aggression towards calves in these interactions does not support infanticide. Rather this may indicate supportive behavior by the males as young members of the social group are extremely vulnerable to predation. Conversely, the males may hedge their bets and stay close to a female with a very young calf in the event that a predator takes the calf and the female becomes receptive. Precocial young and a lack of violent observations does not support paternity confusion as an explanation for multiple mating in the case at hand but it does indicate the possibility of alternative male tending tactics.

The genetic benefits hypothesis proposes several reasons females might mate with multiple males [38]. It is known that multiple mating reduces the proportion of young dead at birth [81, 82] and higher levels of internal relatedness can lead to decreased survival [83, 84] and decreased reproductive success in inbred offspring [41]. Furthermore, animals with higher than normal parental relatedness were more susceptible to illness and post-illness recovery time increased [85]. Among the offspring in our study, we found diverse levels of individual heterozygosity ranging from outbred parents to related parents in the same progeny array. Calf mortality in the study population is approximately 34% [4] and those calves do not survive long enough for genetic sampling. Therefore, we do not have an estimate of individual heterozygosity among failed calves. However, given the low population diversity, females may benefit from even slight increases in genetic variation among her progeny array.

Females that mate with attractive males may pass those genetic characteristics on to male offspring, ensuring sons will be attractive to females and ultimately, successful in reproduction. If all females are attracted to the same characteristics, strong sexual selection on males results. Although sexual size dimorphism occurs in several cetacean species [80], little sexual dimorphism occurs among Atlantic spotted dolphins (e.g., white-tipped rostrum in older males [4]), lending insignificant support the genetic benefits hypothesis. However, females may find non-morphological characteristics such as age or social status as attractive genetic quality indicators. As stated previously, young female Atlantic spotted dolphins preferentially copulate with older males and older males are more often seen attending female-calf pairs [4]. Previous paternity assignments indicated a minimum male age requirement for successful mating [40]. The average male conception age was 25+ years (range 18–30+ years) and the youngest male assigned paternity was 18 years old at the time of conception. Sick and Flyi mated to produce Free in 2001 when Sick was 26+ years old. Sick was 16+ years when KP was conceived, perhaps too young to successfully secure the mating opportunity. Similarly, Bigg was 19+ and 23+ when Brus and Pigm were conceived, potentially supporting the age bias hypothesis. However, our model pedigrees also indicate conception opportunities when the male was older than the average age of successful males (e.g., Bigg was 36+ when Port was conceived), but was not identified as the father of the calf. Therefore, age and long-term viability may be important to receptive females but several additional factors may be significant as well.

In conclusion, our study confirms that females mated with different males over their reproductive life even though the number of suitable males may have been small. The simple explanation of varying male availability during subsequent estrus events cannot explain the observed pattern of multiple mating. Paternity confusion also seems unlikely to explain promiscuous mating among female Atlantic spotted dolphins but alternative tending tactics used by males may occur among the study population. Our data suggests females potentially gain genetic benefits from mating with different males. Because evidence suggests males must reach a minimum age before successfully siring offspring, it is possible that females pass genes for long-term viability to their young. Offspring arrays of females may also benefit from slight increases in genetic diversity but further research is needed. The genetic quality of offspring is not considered a universal factor affecting the development of multi-male mating across all taxa, however, our study presents a species that may garner genetic benefits from promiscuous mating.
